# Low lymphocyte-to-white blood cell ratio and high monocyte-to-white blood cell ratio predict poor prognosis in gastric cancer

**DOI:** 10.18632/oncotarget.14136

**Published:** 2016-12-24

**Authors:** Fan Feng, Li Sun, Gaozan Zheng, Shushang Liu, Zhen Liu, Guanghui Xu, Man Guo, Xiao Lian, Daiming Fan, Hongwei Zhang

**Affiliations:** ^1^ Division of Digestive Surgery, Xijing Hospital of Digestive Diseases, Fourth Military Medical University, 710032, Xi’an, Shaanxi, China

**Keywords:** gastric cancer, blood test, prognosis

## Abstract

Previous results regarding the prognostic value of the neutrophil-to-lymphocyte ratio (NLR) and platelet-to-lymphocyte ratio (PLR) in gastric cancer are conflicting, and full analysis of other blood test parameters are lacking. We therefore examined the associations between various blood test parameters and prognosis in 3243 gastric cancer patients randomly divided into training (n=1621) and validation (n=1622) sets. Optimal cut-off values of 0.663 for neutrophil-to-white blood cell ratio (NWR), 0.288 for lymphocyte-to-white blood cell ratio (LWR), 0.072 for monocyte-to-white blood cell ratio (MWR), 2.604 for NLR, 0.194 for monocyte-to-lymphocyte ratio (MLR), and 130.675 for PLR were identified in the training set. Univariate and survival analyses revealed that high NWR, low LWR, high MWR, high NLR, high MLR, and high PLR are all associated with a poor prognosis in gastric cancer. However, multivariate analysis revealed that only LWR, and MWR are independent prognostic predictors, and prognostic value increased when LWR and MWR were considered in combination. These findings suggest that low LWR and high MWR are each predictive of a poor prognosis, and exhibit greater prognostic value when considered in combination.

## INTRODUCTION

Although incidences have declined in recent decades, gastric cancer is still the fifth most common malignancy and the third leading cause of cancer-related mortality worldwide [1]. Surgical resection with extended lymph node clearance remains the only curative option for non-metastatic gastric cancer. Despite rapid advances in surgical techniques and adjuvant therapy, the prognosis for advanced gastric cancer is still poor [2]. Precise prognostic assessment is critical for optimizing treatment decisions and improving outcomes in gastric cancer patients. [3]. However, accurately predicting outcomes based on the current TNM staging system is difficult because prognosis varies among patients with the same TNM stage. Additional parameters are therefore needed to better identify prognostic factors in patients and to aid in the selection of tailored therapies.

Inflammation is a critical component of tumor progression. Systemic inflammation promotes tumor progression and metastasis by inhibiting apoptosis and promoting angiogenesis [4]. NLR and PLR are simple and cost-effective strategies for evaluating systemic inflammation response and are associated with poor prognosis in various malignancies [5]. Previous reports have also demonstrated that NLR and PLR are associated with outcomes in gastric cancer [6, 7].

Immune response is another important prognostic factor in gastric cancer. Infiltration of tumors by B and Th1 cells is associated with favorable prognosis in gastric cancer patients [8]. High CD3+ levels in peripheral blood cells also predict better survival in gastric cancer patients [9]. Moreover, high frequencies of CD8+ status in CD3+ T cells and of Tregs expression in CD4+ T cells are both correlated with increased survival in gastric cancer patients [10]. Tumor-infiltrating lymphocyte recruitment is also associated with favorable prognosis in advanced gastric cancer [11], and higher numbers of lymphocyte subsets before surgery are associated with better prognosis in gastric cancer [12]. However, the prognostic value of LWR in gastric cancer has not yet been investigated.

In this study, we investigated the prognostic value of various blood test parameters in gastric cancer patients.

## RESULTS

**Figure 1:**
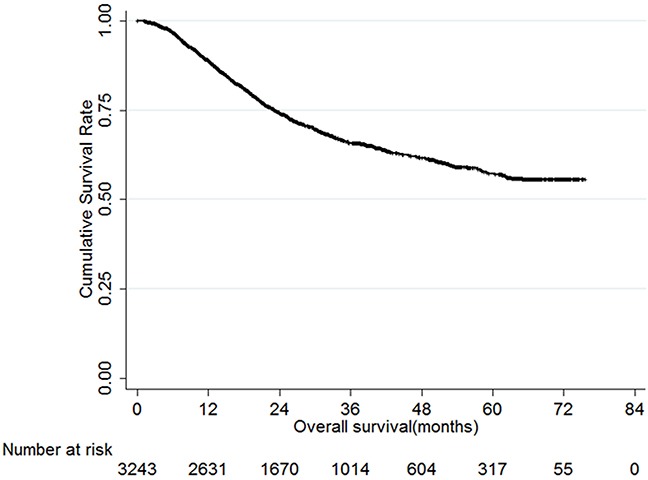
Overall survival of gastric cancer patients

2538 male (78.3%) and 705 female (21.7%) patients between 20 and 90 years old (median, 58; mean, 57.3) were included in this study. Follow up times ranged from 1 to 75 months (median, 24.9; mean, 28.1), and 1-, 3-, and 5-year overall survival rates were 88.9%, 65.8%, and 57.2%, respectively. Figure 1 shows overall survival in these gastric cancer patients.

**Figure 2:**
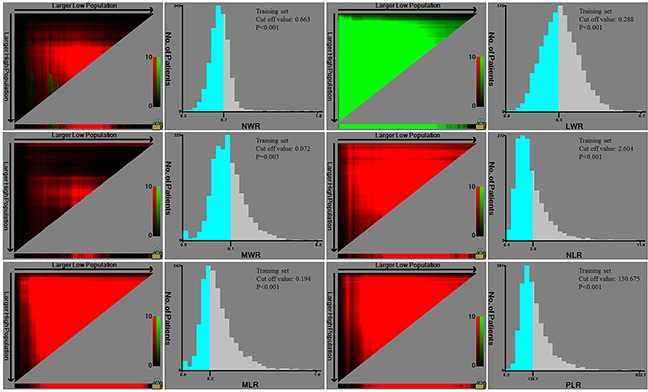
Cut-off values of NLR, MLR, PLR, NWR, LWR, and MWR in training set patients

The training and validation sets consisted of 1621 and 1622 patients, respectively. Clinicopathological features were comparable between the training and validation sets (Table 1). The optimal cut-off value for predicting prognosis in training set gastric cancer patients was 0.663 for NWR (*P<*0.001), 0.288 for LWR (*P*<0.001), 0.072 for MWR (*P*=0.003), 2.604 for NLR (*P*<0.001), 0.194 for MLR (*P*<0.001), and 130.675 for PLR (*P*<0.001) (Figure 2).

**Table 1:** Comparison of clinicopathological characteristics of patients in the training set and validation set

Characteristics	Training setn=1621	Validation setn=1622	P value
Gender			0.695
Male	1264	1274	
Female	357	348	
Age			0.506
≤60	974	956	
>60	647	666	
Tumor location			0.664
Upper third	522	500	
Middle third	256	275	
Lower third	708	721	
Entire	135	126	
Tumor size (cm)			0.667
≤5	1118	1130	
>5	503	492	
Pathological type			0.484
Well differentiated	186	179	
Moderately differentiated	428	399	
Poorly differentiated	911	955	
Signet ring cell or Mucinous	96	89	
Tumor depth			0.336
T1	305	298	
T2	266	233	
T3	578	587	
T4	472	504	
Lymph node metastasis			0.956
N0	579	583	
N1	317	306	
N2	277	285	
N3	448	448	
Tumor stage			0.403
I	406	395	
II	486	460	
III	729	767	

**Figure 3:**
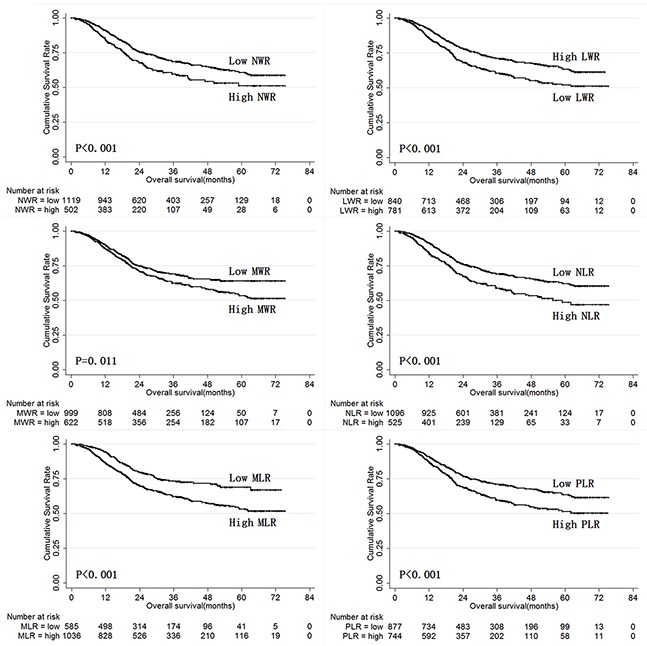
Overall survival of training set gastric cancer patients according to NLR, MLR, PLR, NWR, LWR, and MWR

NLR, MLR, PLR, NWR, LWR, and MWR were therefore examined in univariate and multivariate analysis to identify prognostic predictors in training set gastric cancer patients. Univariate analysis revealed that age, tumor size, pathological type, tumor depth, lymph node metastasis, tumor stage, lymphatic-vascular invasion, neural invasion, NWR, LWR, MWR, NLR, MLR, and PLR were risk factors for gastric cancer prognosis (Table 2). Specifically, high NLR, MLR, PLR, NWR, and MWR (all *P*<0.05) and low LWR (P<0.001) were associated with poor prognosis. Age, tumor size, tumor depth, lymph node metastasis, LWR, and MWR were independent risk factors for prognosis (Table 2). Overall survival in training set gastric cancer patients according to NLR, MLR, PLR, NWR, LWR, and MWR is shown in Figure 3.

**Table 2:** Univariate and multivariate analysis of risk factors for prognosis of gastric cancer patients in training set

Prognostic factors	β	Hazard ratio (95% CI)	P value	β	Hazard ratio (95% CI)	P value
Gender	0.017	1.017(0.818-1.264)	0.878			
Age	0.336	1.399(1.168-1.676)	<0.001	0.262	1.299(1.031-1.638)	0.027
Tumor location	−0.030	0.971(0.910-1.035)	0.363			
Tumor size	1.105	3.020(2.656-3.433)	<0.001	0.487	1.628(1.280-2.070)	<0.001
Pathological type	0.433	1.541(1.414-1.681)	<0.001			
Tumor depth	0.790	2.203(1.970-2.465)	<0.001	0.527	1.694(1.422-2.019)	<0.001
Lymph node metastasis	0.672	1.957(1.801-2.159)	<0.001	0.568	1.764(1.557-1.999)	<0.001
Tumor stage	1.157	3.181(2.727-3.711)	<0.001			
Lymphatic-vascular invasion	1.087	2.966(2.281-3.857)	<0.001			
Neural invasion	1.058	2.879(2.040-4.064)	<0.001			
NWR	0.364	1.439(1.189-1.741)	<0.001			
LWR	−0.397	0.672(0.561-0.806)	<0.001	−0.316	0.729(0.626-0.858)	0.042
MWR	0.236	1.266(1.056-1.518)	0.011	0.272	1.312(1.036-1.662)	0.024
NLR	0.417	1.517(1.260-1.827)	<0.001			
MLR	0.486	1.625(1.322-1.977)	<0.001			
PLR	0.371	1.449(1.210-1.736)	<0.001			

**Figure 4:**
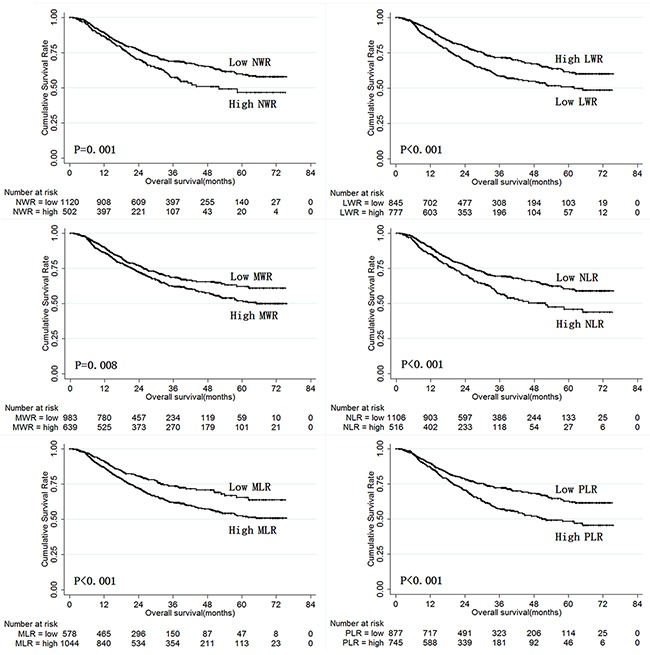
Overall survival of validation set gastric cancer patients according to NLR, MLR, PLR, NWR, LWR, and MWR

The prognostic value of NLR, MLR, PLR, NWR, LWR, and MWR was also analyzed in the validation set patients using the cut-off values established in the training set (Table 3). The results obtained in validation set patients were similar to those obtained in the training set, and LWR and MWR were again identified as independent risk factors for prognosis in validation set patients. Overall survival in validation set gastric cancer patients according to NLR, MLR, PLR, NWR, LWR, and MWR is shown in Figure 4.

**Table 3:** Univariate and multivariate analysis of risk factors for prognosis of gastric cancer patients in validation set

Prognostic factors	β	Hazard ratio (95% CI)	P value	β	Hazard ratio (95% CI)	P value
Gender	0.128	1.137(0.916-1.410)	0.244			
Age	0.181	1.198(1.000-1.436)	0.005	0.223	1.250(1.000-1.562)	0.050
Tumor location	0.008	1.008(0.919-1.105)	0.869			
Tumor size	1.057	2.879(2.400-3.453)	<0.001	0.283	1.327(1.045-1.684)	0.020
Pathological type	0.415	1.515(1.340-1.711)	<0.001			
Tumor depth	0.799	2.224(1.983-2.494)	<0.001	0.453	1.573(1.317-1.880)	<0.001
Lymph node metastasis	0.750	2.117(1.944-2.305)	<0.001	0.550	1.734(1.533-1.962)	<0.001
Tumor stage	1.329	3.776(3.191-4.468)	<0.001			
Lymphatic-vascular invasion	1.174	3.234(2.475-4.226)	<0.001			
Neural invasion	1.389	4.012(2.699-5.964)	<0.001	0.453	1.574(1.031-2.403)	0.036
NWR	0.329	1.389(1.147-1.682)	0.001			
LWR	−0.426	0.653(0.545-0.783)	<0.001	−0.389	0.678(0.540-0.851)	0.001
MWR	0.247	1.281(1.068-1.536)	0.008	0.334	1.397(1.112-1.755)	0.004
NLR	0.396	1.486(1.232-1.791)	<0.001			
MLR	0.410	1.506(1.226-1.851)	<0.001			
PLR	0.435	1.545(1.289-1.852)	<0.001			

**Figure 5:**
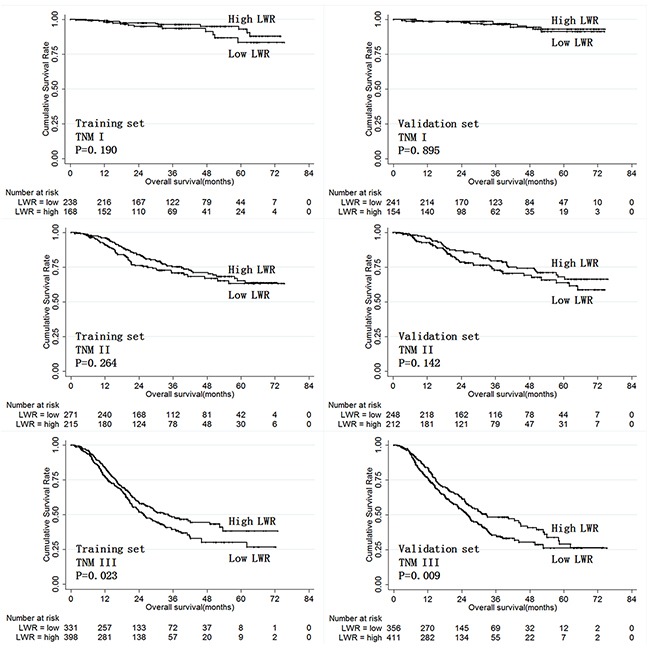
Overall survival according to LWR in training and validation set patients stratified by TNM stage

**Figure 6:**
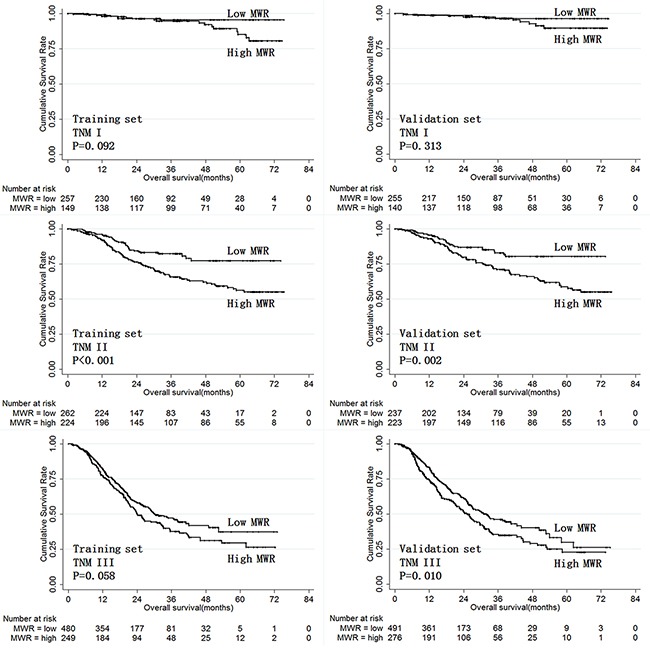
Overall survival according to MWR in training and validation set patients stratified by TNM stage

Next, we analyzed the prognostic value of LWR and MWR in the both the training and validation sets when patients were stratified by TNM stage. While LWR was only predictive of prognosis in stage III gastric cancer patients (Figure 5), MWR was predictive of prognosis in both stage II and III gastric cancer patients (Figure 6).

**Figure 7:**
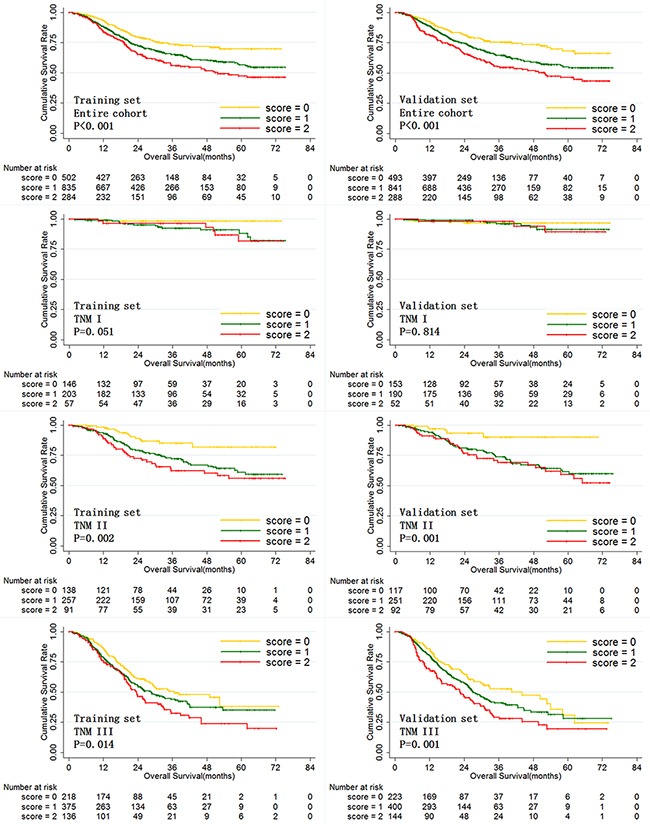
Overall survival of training and validation set gastric cancer patients according to LWR and MWR in combination

Finally, we evaluated the prognostic value of LWR and MWR in combination in both training and validation set gastric cancer patients. Patients with high LWR and low MWR were given a score of 0. Patients with high LWR and high MWR or with low LWR and low MWR were given a score of 1. Patients with low LWR and high MWR were given a score of 2. Figure 7 shows overall survival depending on score for the three score groups in training and validation set patients. Overall survival decreased as scores increased both in the training and validation sets individually and for the entire patient cohort combined. Moreover, LWR and MWR in combination were predictive of prognosis in both stage II and III gastric cancer patients.

## DISCUSSION

Blood tests are simple, convenient, reproducible, and cost-effective. However, the prognostic value of NLR and PLR in gastric cancer remains controversial, and full analyses of the prognostic values of NLR, MLR, PLR, NWR, LWR, and MWR are lacking. Therefore, in the present study we investigated the prognostic value of these blood test parameters in gastric cancer patients. This is the largest analysis of associations between blood test results and gastric cancer prognosis to date. We found that high NLR, MLR, PLR, NWR, and MWR and low LWR were all associated with poor prognosis in gastric cancer patients. However, only LWR and MWR were independent prognostic factors specifically in stage III and stage II/III gastric cancer patients, respectively. Moreover, LWR and MWR in combination improved prognosis prediction in stage II/III gastric cancer patients.

Peripheral neutrophil is a marker of acute and chronic inflammation [13], and upregulation of peripheral neutrophils in response to the production of hematopoietic cytokines by tumor cells is indicative of more aggressive tumors [14]. Here, we found that high NWR was associated with poor prognosis in gastric cancer patients. Previous reports have shown that neutrophils promote tumor growth and metastasis by secreting vascular endothelial growth factor [15], chemokines [16] and matrix metalloproteinase-9 [17]. Neutrophils also increase adhesion between end organs and circulating tumor cells, thus increasing the chances of metastatic seeding. Spicer *et al*. reported that neutrophils act as adhesive adapters between circulating tumor cells and metastatic targets [18]. In addition, elevated neutrophil levels in the vicinity of tumors may inhibit the antitumor effects of activated T cells and natural killer cells [19].

Lymphocytes play critical roles in host immune responses and possess potent anticancer activities that inhibit growth and metastasis in several tumors [20]. Furthermore, increased lymphocyte levels are associated with better prognosis in a variety of tumors [21]. Consistent with these results, we found here that high LWR was associated with better prognosis in gastric cancer patients.

NLR, the most widely-used parameter for predicting prognosis in gastric cancer, reflects the balance between pro-tumor inflammatory status and anti-tumor immune status. Increased NLR, which may reflect increased inflammation and/or decreased immune reaction, is associated with poor prognosis in various tumors [5]. Two meta-analysis studies have demonstrated that elevated NLR is associated with poor prognosis in gastric cancer as well [6, 22]. We also found here that NLR was associated with prognosis in gastric cancer. However, NLR was not an independent prognostic factor. The inclusion of six blood test parameters in our univariate and multivariate analysis might explain this inconsistency between our study and previous reports.

Platelets also play a critical role in tumor development and progression, and thrombocytosis is associated with poor prognosis in a variety of solid tumors [6]. Platelets promote tumor growth by increasing angiogenesis via cytokine vascular endothelial growth factor [23] and protect tumor cells against environmental stresses in the blood stream, including immune attack, shear force, and mechanical trauma [24]. Moreover, platelets promote tumor chemotaxis, adhesion, proliferation, and metastatic spread through extensive interactions with tumor cells [25]. For these reasons, PLR has been extensively investigated and is a valuable prognostic factor for several solid malignancies [5]. Here, we found that PLR was also associated with prognosis in gastric cancer, although it was not an independent prognostic factor.

Although the underlying mechanisms remain unknown, monocyte levels are also associated with prognosis in a variety of tumors, including gastric cancer [26]. In the present study, we found that high MWR and MLR were both associated with poor prognosis in gastric cancer. Moreover, MWR was identified as an independent prognostic factor. Monocytes promote angiogenesis and tumorigenesis [27] and suppress host anticancer immune responses, which may explain why elevated monocyte levels were associated with poor prognosis [28]. Monocytes also promote the development of malignant cells by secreting soluble mediators [29]. In addition, cytokines and chemokines produced by tumor cells can trigger the differentiation of monocytes into tumor-associated macrophages [30], which in turn promote tumor cell growth, migration, and metastasis [31].

While treatments that specifically modify pre- and post-operative imbalances between inflammatory and immune status might improve long-term outcomes for patients with malignant tumors, no such therapies currently exist. In addition, inflammatory and immune response parameters might help predict responses to and toxicity resulting from different treatments; future studies are needed to examine these possibilities.

Several limitations of the present study should be considered when interpreting the results. First, it was a retrospective study of patients from a single institution, and multi-center studies are needed to confirm the predictive value of the parameters identified here. Second, the cut-off values used in the present study differed from values used in our previous studies; standard cut-off values that are effective in prospectively predicting gastric cancer prognosis across studies should be identified. Third, the prognostic value of absolute differential white cell counts in gastric cancer patients was not examined here [32].

In conclusion, high NLR, MLR, PLR, NWR, and MWR and low LWR were all associated with poor prognosis in gastric cancer patients. However, only LWR and MWR were identified as independent prognostic factors specifically in stage III and stage II/III gastric cancer patients, respectively. Moreover, LWR and MWR combined was the best predictor of prognosis in stage II/III gastric cancer patients.

## MATERIALS AND METHODS

This study was performed at the Xijing Hospital of Digestive Diseases affiliated with the Fourth Military Medical University. 3243 gastric cancer patients were enrolled in the study between September 2008 and March 2015. Patient inclusion criteria were as follows: 1. no other malignancy, 2. no distant metastasis, 3. no history of neoadjuvant chemotherapy, 4. had undergone radical D2 gastrectomy, 5. preoperative blood tests were available, 6. no signs of infection, 7. follow-up data were available. This study was approved by the Ethics Committee of Xijing Hospital, and written informed consent was obtained from all patients before surgery.

Preoperative blood tests were performed within 7 days prior to surgery. Blood NLR was calculated by dividing neutrophil count (number of neutrophils/μL) by lymphocyte count (number of lymphocytes/μL). MLR, PLR, NWR, LWR, and MWR were calculated in the same way as NLR using the corresponding cell counts.

Clinicopathological data, including gender, age, blood test results, tumor location, tumor size, pathological type, tumor depth, lymph node metastasis, tumor stage, lymphatic-vascular invasion, and neural invasion were collected. Follow-ups involving enhanced chest and abdominal CT and gastroscopy were conducted every 3 months until November 2015.

Data were processed using SPSS 22.0 for Windows (SPSS Inc., Chicago, IL, USA). X-tile software [33] was used to randomly assign gastric cancer patients to the training and validation sets with a sample size ratio of 1:1. Optimal cut-off values for preoperative NLR, MLR, PLR, NWR, LWR, and MWR for predicting gastric cancer prognosis were calculated using X-tile software. Discrete variables were analyzed using Chi-square tests or Fisher's exact tests. Significant risk factors for prognosis in gastric cancer patients identified by univariate analysis were further assessed with multivariate analysis using the Cox's proportional hazards regression model. Overall survival was analyzed using the Kaplan-Meier method. *P* values of less than 5% were considered statistically significant.
